# First principles study of the stability of MXenes under an electron beam†

**DOI:** 10.1039/d0na00886a

**Published:** 2021-02-17

**Authors:** Rina Ibragimova, Zhong-Peng Lv, Hannu-Pekka Komsa

**Affiliations:** Department of Applied Physics, Aalto University 00076 Aalto Finland; Microelectronics Research Unit, University of Oulu 90014 Oulu Finland hannu-pekka.komsa@oulu

## Abstract

Interactions between two-dimensional MXene sheets and electron beams of a (scanning) transmission electron microscope are studied by first-principles calculations. We simulated the knock-on sputtering threshold for Ti_3_C_2_ MXene sheets *via ab initio* molecular dynamics simulations and for five other MXenes (Ti_2_C, Ti_2_N, Nb_2_C, Mo_2_TiC_2_, and Ti_3_CN) approximately from defect formation energies. We evaluated the sputtering cross section and sputtering rates and based on those evaluated the surface composition. We find that at the exit surface and for “low” TEM energies H and F sputter at equal rates, but at “high” TEM energies the F is sputtered most strongly. In the entry surface, H sputtering dominates. The results were found to be largely similar for all studied MXenes, and although the sputtering thresholds varied between the different metal atoms the thresholds were always too high to lead to significant sputtering of the metal atoms. We simulated electron microscope images at the successive stages of sputtering and found that while it is likely difficult to identify surface groups based on the spot intensities, the local contraction of the lattice around O groups should be observable. We also studied MXenes encapsulated with graphene and found them to provide efficient protection from knock-on damage for all surface group atoms except H.

## Abstract 2

Interactions between two-dimensional MXene sheets and electron beams of a (scanning) transmission electron microscope are studied by first-principles calculations.

## Graphical abstract

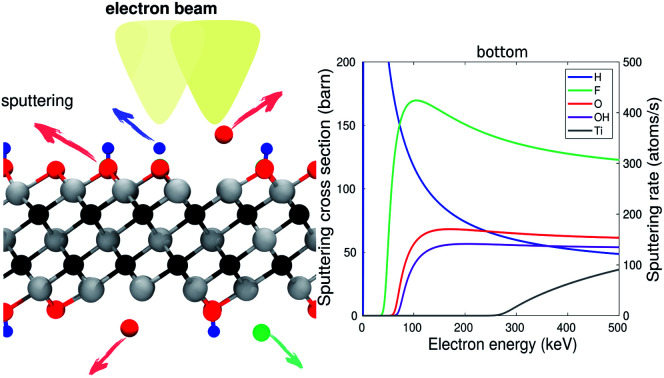

## 1. Introduction

MXenes are a class of two-dimensional materials of transition metal carbides and nitrides, with the chemical formula M_*n*+1_X_*n*_T_*x*_.^1–3^ These materials are obtained *via* selective etching of layered bulk precursor phases using, *e*.*g*., hydrofluoric acid (HF),^3,4^ which results in the passivation of surface sites by functional groups T_*x*_ from the solution, where T_*x*_ predominantly consists of O, OH, and F. MXenes possess many beneficial properties, such as good electrical conductivity, hydrophilicity, flexibility, mechanical strength, consisting of abundant elements, stability in solution and the ease of synthesis in large batches. In particular, the combination of these advantages has made them suitable for many applications such as batteries, supercapacitors, electromagnetic interference shielding, sensors, and wearable devices.^2,5–8^ Importantly, the surface functional groups as well as defects in the MX backbone can have a significant effect, either beneficial or detrimental, on the material properties. Thus a lot of effort has been devoted to studying them *via* XPS, NMR, X-ray and neutron scattering, Raman spectroscopy, and (scanning) transmission electron microscopy [(S)TEM]. (S)TEM appears particularly suited for the study of defects as it can provide direct structural information and has been successfully employed for other 2D materials.^9–12^

(S)TEM images of the defects on Ti_3_C_2_ MXenes were reported in ref. 13–16, and mostly show Ti-related defects: vacancies and adatoms. Unfortunately, C, O, F, and H atoms are difficult to identify reliably, since the STEM signal is proportional to the atomic number as ∼*Z*^1.7^, and thus Ti atoms dominate the signal. While the O, OH, and F atoms cannot be seen directly, the brighter areas were assigned to regions with a higher concentration of O atoms, which seemed to agree with EELS.^13,15^ In addition, Sang *et al.*^14^ observed correlation between the etching conditions and vacancy concentration, and also clustering of vacancies.

Interactions between a relativistic electron and a sample can lead to damage *via* several different mechanisms.^17^ Elastic collision between an electron and nucleus is called the knock-on mechanism. In inelastic collision, energy is lost to electronic excitation leading to direct bond breaking (radiolysis), heating, or charging. In addition, the beam can crack gas molecules or contaminants on the surface, and these radicals can lead to chemical etching.^18^ In the case of graphene, knock-on dominates and heating and charging effects can be ignored due to very high electrical and thermal conductivity. In the case of BN, the charging effects can be significant. Radiolysis is known to be important for organic compounds, but plays a minor role in conducting samples due to a short excitation lifetime. Semiconductors, such as TMDs, fall somewhere in between.^12,19^ Also, in the case of MXenes, due to their high electrical conductivity, we expect radiolysis and charging effects to play a minor role. The thermal conductivity is also reasonably high. In the knock-on mechanism, heavier atoms are more stable under the beam. All atoms in Ti_3_C_2_T_*x*_ are relatively light and thus susceptible to sputtering. In ref. 16, Sang *et al.* studied Ti dynamics and observed Ti displacement and hole growth, but also the formation of thicker Ti_*n*+1_C_*n*_ layers. According to Zhang *et al.*,^20^ prolonged irradiation parallel to the layers led to the removal of H and “repartitioning” of Ti and O atoms between MXene layers. Although lighter atoms are expected to be sputtered, since they are not directly seen, the microscopic details have remained elusive. To this end, atomistic simulations could prove highly useful in providing the missing details.

Here, we present a first principles study on the stability of MXenes under an electron beam. We carried out *ab initio* molecular dynamics (AIMD) simulations to determine the sputtering thresholds and sputtering cross sections of functional groups and Ti atoms of Ti_3_C_2_T_*x*_ monolayers. We focus on the knock-on mechanism, since we expect it to dominate for the above-mentioned reasons, but also because it is straightforward to simulate reliably using first principles simulations. Based on this, we can estimate the order (and rate) in which each functional group is removed from the top and bottom surfaces. We also simulate (S)TEM images for the structures at successive stages of sputtering. For several other MXenes, we evaluate the sputtering thresholds using unrelaxed vacancy formation energies, since AIMD is computationally demanding and this approximation is found to work well. We also consider protecting the MXene sheets by sandwiching them with graphene that has been found to be resistant to damage from electron irradiation. Finally, the results are compared to the existing literature.

## 2. Methods

Density-functional theory calculations were used to model the electron-beam interaction with single MXene sheets. All calculations were performed using the projector augmented wave formalism as implemented in the simulation package VASP.^21,22^ For all calculations we adopt the Perdew–Burke–Ernzerhof for solids (PBEsol) exchange correlation functional,^23^ as it was found to well reproduce the heats of formation of relevant compounds.^24^ For the heterostructure with graphene, we keep PBEsol in order to have consistent description of the electronic structure but we also include DFT-D3 van der Waals corrections to improve the interlayer binding.^25^ The optimal plane-wave cutoff energy was chosen as 500 eV according to the convergence test. A *k*-points set of 3 × 3 × 1 was chosen as optimal for monolayer calculations with a supercell size of 4 × 4 × 1.

As we found previously in the case of Ti_3_C_2_, the composition of surface functional groups at a given pH and work function can be a mixture of F, O, and OH.^24^ Here, we employ a 4 × 4 supercell special quasi-ordered structures with a composition O_0.5_F_0.25_OH_0.25_, as constructed in ref. 24. The heterostructure of Ti_3_C_2_-graphene was constructed by placing the 4 × 4 MXene supercell on a 5 × 5 supercell of graphene and the lattice constant was fixed to that of the MXene sheet. Defects in the other MXene sheets are also modeled using a 4 × 4 supercell.

**Fig. 1:**
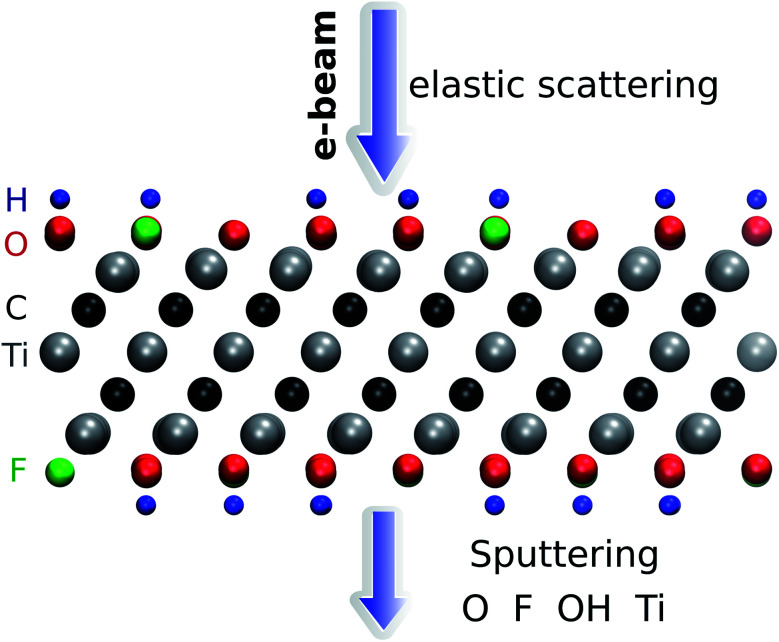
Illustration of the simulation setup used for *ab initio* MD calculations of Ti_3_C_2_(O_0.5_F_0.25_OH_0.25_)^2^. The adopted convention for the direction of the electron beam is also shown.

The threshold energy for sputtering atoms was determined by running a series of *ab initio* molecular dynamics (AIMD) simulations. Assuming that momentum transfer from the electron to the atom is instantaneous and the collision is fully elastic, we can then use the energy and momentum conservation principle. The initial kinetic energy transferred to the atom is increased until we find a minimal energy needed to sputter the atom from the lattice. The calculations are performed with a step of kinetic energy of 0.1 eV for those processes with a low sputtering threshold and with a step of 1 eV for those with a high sputtering threshold (above 20 eV). The computational setup is illustrated in Fig. 1. We adopt a convention where the electrons enter from the top and exit from the bottom of the sheet. We used a small time step of 0.5 fs for most of the molecular dynamics calculations and a 0.1 fs timestep for modelling H sputtering. All reported results are for the initial velocity of the ion parallel to the electron beam, *i.e.*, normal to the MXene sheet, corresponding to the maximum transferred energy. For the atoms at the top surface, we also carried out MD simulations at selected oblique angles, but this always yielded higher sputtering thresholds. Further details are given in the ESI.†^28^

Sputtering cross sections are calculated using the McKinley–Feshbach formalism,^26^ which is generally valid (*i*.*e*., equal to the Mott formula) at *Z* < 29 and thus valid for all atoms in our Ti_3_C_2_T_*x*_ systems. The effect of finite ion velocities is accounted for as described in ref. 27 and by adopting the Maxwell–Boltzmann velocity distribution.

The sputtering rate of atoms can be found by multiplication of cross sections by a current density of a microscope, assuming that 1 electron sputters out 1 atom, *S* = *σJ*. The reported electron microscopy studies of MXenes^13–15,15,20^ have used electron energies 60, 80, 100, and 300 keV and beam currents of 10–100 pA, where reported. Usually larger current is needed at lower electron energies. Assuming STEM configuration with a beam current of 15 pA focused on an area of one unit cell (or one surface site, about 9 Å^2^), we obtain a proportionality factor of 0.1, *i*.*e*., cross section of 100 barn gives a sputtering rate of about 10 atoms per s. We expect that our estimate for the rate is likely on the lower bound. The rate equations for determining the evolution of composition with time are given in the ESI.†^28^

For the other MXenes, the sputtering threshold is evaluated only using the unrelaxed defect formation energy approach, which was found to work well in the case of “rigid” 2D materials and when the sputtered atom's trajectory is unobstructed.^12^

STEM image simulations were carried out using DrPROBE software^29^ at acceleration energies of 60 and 300 keV, and using a probe size of 0.04 nm (half-width-half-maximum).^14,30^ Other simulation parameters are a convergence semi-angle of 21.5 mrad, spherical aberration of 200 nm, chromatic aberration of 1.5 mm, 3-fold astigmatism of < 30 nm, axial coma of < 30 nm, 4-fold astigmatism of 500 nm, star aberration of 500 nm, high-angle annular dark-field (HAADF) inner detector angle of 80 mrad, HAADF outer detector angle of 250 mrad, ABF (annular bright field imaging) inner detector angle of 12 mrad, and ABF outer detector angle of 24 mrad.

## 3. Results

### 3.1. Ti_3_C_2_ under an electron beam

**Fig. 2:**
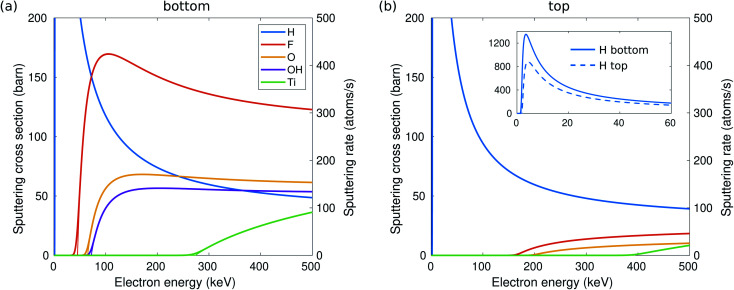
Sputtering cross section for H, F, O, OH, and Ti and the corresponding sputtering rates depending on TEM electron energy from the bottom (a) and top (b) surfaces of Ti_3_C_2_(O_0.5_F_0.25_OH_0.25_)^2^. The thick lines include the effect of thermal vibrations, whereas the thin lines correspond to the static lattice. The inset shows enlarged H cross sections at low electron energies.

The calculated sputtering threshold energies, as well as the corresponding electron energies, are given in Table 1 and in Fig. 6. We have considered sputtering of O and F ions in the case of O and F functional groups, both O and H separately in the case of the OH group (denoted respectively as OH and H in Table 1), and Ti atoms in the bare (unterminated) surface. As seen in Fig. 2, the effect of thermal vibrations on the cross sections is very small.

**Table 1:** Calculated sputtering threshold energy (transferred from electrons to atoms) *T*_k_ and the corresponding TEM acceleration voltage *E*_el_ for the top and bottom surfaces of Ti_3_C_2_(O_0.5_F_0.25_OH_0.25_)^2^

	Top		Bottom	
	*T*_k_ (eV)	*E*_el_ (keV)	*T*_k_ (eV)	*E*_el_ (keV)
O	33	200	9.5	65
F	22	165	5.5	46
H	4.7	2.2	3.8	1.7
OH	32	200	10.6	72
Ti	25	391	15.9	270

First focusing on the bottom side, threshold energies for the sputtering of functional groups and consequently the electron energies are rather small: H sputters out from the surface at above 1.7 keV, F is sputtered at 46 keV, O at 68 keV, and finally OH at 72 keV. The threshold energies were found not to vary significantly with composition. Slightly lower energies were obtained for sputtering atoms from the pure terminated surface (*e*.*g*., about 9.2 eV for the pure O-terminated surface) and higher in the case of O_0.5_F_0.25_OH_0.25_ termination. We stress that the reported values correspond to the sputtering of atoms. Various other processes may take place below the sputtering threshold depending on the neighborhood of the sputtered atom(s). In the case of O_0.5_F_0.25_OH_0.25_ or pure surface termination, the nearly sputtered atoms fall back to the same position of the surface, mainly since we provide only momentum perpendicular to the surface. In the case of O_0.5_OH_0.5_, the O atom falling back to the surface can capture a H atom from the neighboring OH sites, *i*.*e*., leading to the diffusion of H, or, at just below the sputtering threshold, even bond with two H atoms and form a water molecule, which can desorb from or migrate on the surface.

On the other hand, Ti atoms (on a bare surface) are stable under the beam, up to 270 keV, and even then the sputtering rate is relatively low. Next to the Ti vacancy, the threshold drops to 12 eV, but still relatively high. This is consistent with the experimental observations of the Ti_3_C_2_ sheets.^13–16^

On the top surface, sputtering thresholds are much higher for all cases except H. The dominant sputtering process in our simulations is a straightforward “bounce” from the Ti_3_C_2_ backbone. This is caused by the fact that we only carried out simulations with on-axis collisions, *i*.*e*., initial momentum perpendicular to the surface. We speculate that in the case of off-axis collision, the probability for *e*.*g*. the formation of water or migration of functional groups is higher on the top surface than on the bottom surface. The formation of water is an important aspect of MXenes, since many experimental studies report water presence between the layers even after drying.^31–33^

For a more quantitative estimate of the sputtering probabilities and the resulting surface composition, we first calculated the sputtering cross sections, as shown in Fig. 2. Although sputtering H requires little kinetic energy and thus dominates at low electron energies, the cross section at larger electron energies becomes smaller due to the low atomic number. At electron energies above 80 keV, F will be sputtered faster than H and with a probability about twice that for O. The sputtering rates are shown on the right axis in Fig. 2(a). Only at 60 kV, some surface groups would remain at the bottom surface, but at >100 kV all functional groups should be quickly (<1 s) sputtered out and the bottom surface becomes bare.

**Fig. 3:**
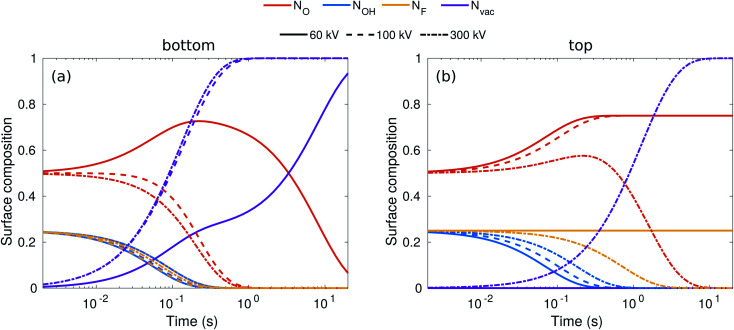
Evolution of surface composition with time on the bottom (a) and top (b) surfaces for three typical acceleration voltages.

While the evolution of the surface composition at low voltages is clear, at 300 kV, sputtering rates for O, OH, and H are all similar and thus the composition evolution is less obvious. To this end, we solved the rate equations (see the ESI†).^28^ The results for 60, 100, and 300 kV electron energies are shown in Fig. 3. At 60 kV, the rapid H sputtering leads to the conversion of all OH groups to O groups at both the top and bottom surfaces. In addition, F groups are quickly sputtered from the bottom surface, but not from the top. At *t* ≈ 1 s, the composition is close to O_0.75_ at the bottom surface and O_0.75_F_0.25_ at the top surface. This is followed by gradual O sputtering from the bottom surface, eventually leading to a bare bottom surface, whereas the top surface remains covered by O and F.

At 100–300 kV, all surface groups are sputtered rapidly from the bottom surface, leading to a bare surface already at *t* = 1 s. On the top surface, 100 kV behavior is similar to 60 kV, but at 300 kV both F and O start sputtering out, although at a lower rate than from the bottom surface, leading to a bare top surface at about *t* = 10 s.

Overall, the sputtering rates are very high and thus we expect that the bottom surface will quickly become clear of surface groups and also the top surface under certain conditions. However, a bare MXene surface is very reactive meaning that any residual gases in the chamber are likely to stick very effectively on it and thereby “refunctionalizing” it. For instance, it was computationally predicted that when a water molecule adsorbs to a bare MXene surface it dissociates into OH and H with a low barrier.^34^ Ambiguous functionalization naturally impedes the interpretation of the obtained images. On the other hand, if a bare surface can be obtained, one could imagine exploiting it to refunctionalize the surface with, *e.g.*, chalcogen and halogen group atoms^35,36^ or even CO_2_.^37,38^

### 3.2. Surface group identification in TEM

According to our results in the previous section, while the bottom surface is expected to become clear of functional groups rather rapidly, at small doses the top surface should remain functionalized. We simulated the STEM images for the successive stages of the sputtering of functional groups for the scenarios of a low electron energy of 60 kV and a high electron energy of 300 kV.

**Fig. 4:**
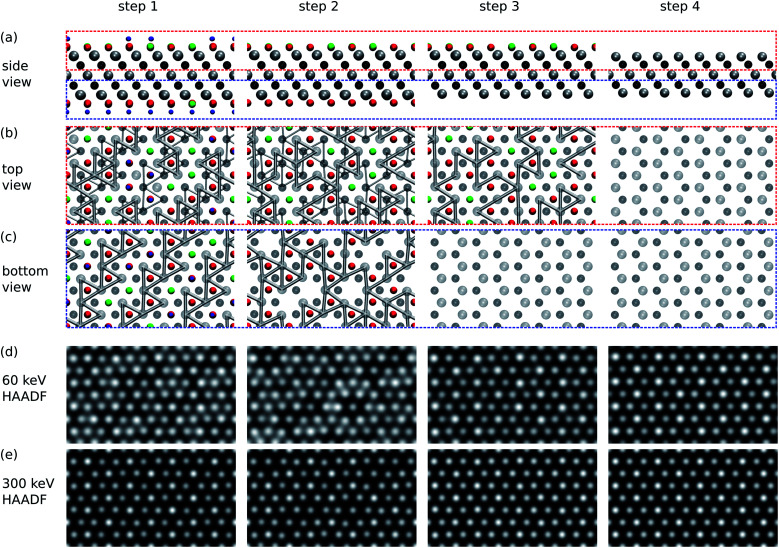
Simulated STEM images of Ti_3_C_2_(O_0.5_F_0.25_OH_0.25_)^2^ at successive stages of functional group sputtering. Step 1 corresponds to the initial fully covered surface, steps 2 and 3 to partially covered surfaces, and step 4 to bare surfaces. See the text for full description. (a) Side views of the structure. Atoms are colored as in Fig. 1. (b) Top views of the top part of the sheet [red box in (a)]. Only Ti–Ti bonds shorter than 3 Å are shown. Thin bonds are those from the bottom part of the sheet. (c) Bottom views of the bottom part of the sheet. Only bonds for the bottom part are shown. (d and e) Simulated HAADF STEM images for 60 keV (d) and 300 keV (e) acceleration voltages.

The results are collected in Fig. 4, where we show the images for four cases: (1) the initial state with a fully functionalized surface, (2) the surface after 1 s of 60 kV irradiation leading to a bottom surface composition of O_0.75_ and top surface composition of O_0.75_F_0.25_, (3) the surface after 10 s of 60 kV irradiation leading to a bare bottom surface and top surface composition of O_0.75_F_0.75_, and (4) the surface after 10 s of 300 kV irradiation leading to two bare surfaces.

We first focus on the images simulated for the 300 kV acceleration voltage. In step 1, the sites with two functionalized groups and one Ti atom per column show up brighter than the other two sites with only one Ti and one C atom per column. One cannot distinguish between O, OH, and F groups from the intensities. The image from step 2 shows distinguishable intensity differences between functionalized and unfunctionalized sites on the bottom surface, but in step 3 the functionalized and unfunctionalized sites cannot be distinguished on the top surface. Note that step 4 with two bare surfaces still shows brighter spots, but not on the sites of surface functionalization as in steps 1 and 2. Instead, the bright spots occur at sites in which Ti atoms are located on the top surface and show that the image contrast depends on the beam propagation direction and on the focus. The corresponding ABF images and a focus series are shown in the ESI.†^28^

Images simulated for the 60 kV acceleration voltage, in steps 1 and 2, show areas with darker and brighter contrast. These features do not arise from the brightness of the spots, but rather from the relative distance between the spots. That is, the bright areas contain O, which locally contracts the lattice around it and leads to shorter Ti–Ti bonds, as illustrated in the top and bottom views of Fig. 4. In the 60 kV and 300 kV images the underlying structure is the same, but due to the larger extent of the spots in the 60 kV images, the relative distances are visually accentuated. On the other hand, this masks the brightness variations between the functionalized and unfunctionalized surfaces, which are perhaps easier to distinguish in the 300 kV images. In steps 3 and 4 of 60 kV images, one again mostly sees the top-most Ti layer.

Given the experimental noise and any extraneous species adsorbed on the MXene surfaces, the identification of the surface groups is likely much more difficult than suggested by these simulated images. Nevertheless, we think that the Ti–Ti bond contraction around O groups should be observable under the right imaging conditions and/or following suitable image processing, thus providing information about the composition and distribution of O on the MXene surfaces.

### 3.3. Protecting Ti_3_C_2_ with monolayer graphene

We also investigated the possibility of protecting the MXenes from beam damage by sandwiching them between graphene layers. Such a strategy has been successfully employed to protect other 2D materials, such as transition metal dichalcogenides and black phosphorus.^19,39,40^

**Fig. 5:**
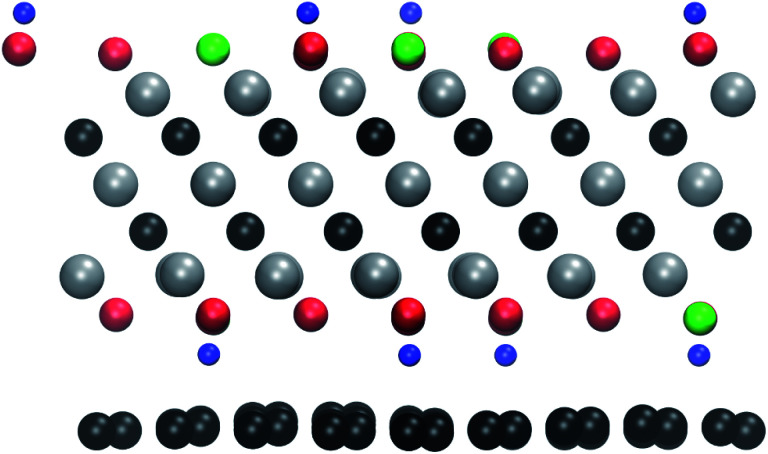
The relaxed atomic structure for the Ti_3_C_2_(O_0.5_F_0.25_OH_0.25_)^2^-graphene heterostructure. The atoms are colored as in Fig. 1.

The relaxed atomic structure is shown in Fig. 5. The MXene layer remains flat, but there are pronounced corrugations in the graphene layer. They arise from the different interactions with the surface groups, *i*.*e*., a shorter interlayer distance in the case of OH groups and larger in the case of O and F groups. Since these calculations become computationally much more demanding, we here carry out the simulations for only one electron energy, namely 100 keV, and only for the bottom surface. As can be seen in Table 1, at 100 keV all functional groups are expected to sputter easily from the unprotected surface.

The graphene protects the bottom surface rather well from sputtering events. At 100 keV all other surface groups remain stable, except H, which can be sputtered from the surface through the graphene lattice. This is not surprising, given that the maximum transferred kinetic energy from 100 keV electrons to H atoms is about 240 eV and the calculated energy barrier for atomic H penetrating graphene is only 2.6–4.6 eV.^41–43^ With such contrast of energies, it seems likely that H atoms could also be sputtered through the graphene-protected top surface. Thus, a heterostructure of MXenes without any OH groups and graphene could be obtained by irradiating the sandwich structure with an electron beam. On the other hand, the energetic O, H, and F atoms impinging on the graphene may react with it, especially if defects are present, and lead to the formation of (fluorinated) graphene oxide.^44^

### 3.4. Evaluated thresholds for other MXenes

**Fig. 6:**
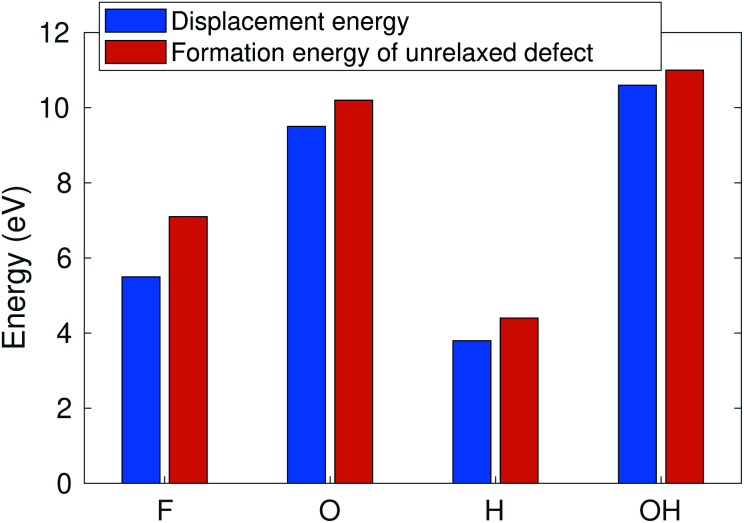
Sputtering threshold energy of the Ti_3_C_2_(O_0.5_F_0.25_OH_0.25_)^2^ surface *vs.* the formation energy of unrelaxed defects.

According to ref. 12, the defect formation energies for unrelaxed defects are close to sputtering threshold kinetic energies from MD calculations in the case of “rigid” 2D materials. To verify that this holds true also for MXenes, we compare in Fig. 6 the AIMD calculated sputtering thresholds with the defect formation energies. The formation energy is consistently a bit higher (0.5–1 eV) than the sputtering threshold, reflecting that a small part of the energy is deposited on the host. Note that this approximation only works for the bottom atoms for which the sputtering trajectory is unobstructed. This allows us to qualitatively evaluate the threshold energies for many more MXene systems.

The calculated defect formation energies for several distinct MXene systems such as Ti_3_C_2_, Ti_2_N, Nb_2_C, Mo_2_TiC_2_, and Ti_3_CN are given in Table 2. In this case, the metal atom vacancies are created in the bare surface, and O, F, and H vacancies in the pure O-, F-, and OH-covered surfaces. Carrying out the whole procedure to determine the surface group composition and distribution for all different materials is beyond the scope of this paper. The results are very similar for all Ti-containing MXenes. In the case of Nb_2_C and Mo_2_TiC_2_, the metal atom sputtering threshold is somewhat higher and the O and F thresholds are somewhat lower, but still differing by only about 1 eV. From this, we conclude that bonding strength with surface groups is similar, and consequently the sputtering under an electron microscope should be similar for all MXenes, *i*.*e*., to the first approximation, our results for Ti_3_C_2_ should also be valid for other MXenes. It is also worth reminding that since Mo and Nb atoms are also clearly heavier than Ti, the transferred kinetic energy is lower and therefore the corresponding electron energy is much higher. Consequently Nb and Mo sputtering should be unlikely under typical acceleration voltages.

**Table 2:** Formation energies for unrelaxed defects *E*_f_ (in eV), which is here used to approximate *T*_k_, for Ti_3_C_2_, Ti_2_N, Nb_2_C, Mo_2_TiC_2_, and Ti_3_CN. M refers to the outer metal atom, which is Mo in the case of Mo_2_TiC_2_

	Ti_3_C_2_	Ti_2_C	Ti_2_N	Nb_2_C	Mo_2_TiC_2_	Ti_3_CN
M	11.4	11.4	10.8	14.0	15.3	11.0
O	10.2	10.2	10.1	9.8	8.7	10.0
F	6.4	6.4	6.2	5.0	4.9	6.4
H	3.1	3.0	3.0	2.8	3.1	3.0

## 4. Conclusions

We have carried out first principles calculations to study the stability of MXenes under an electron beam. In particular, the threshold energies for the sputtering of surface group atoms *via* the knock-on mechanism were evaluated using *ab initio* molecular dynamics simulations. We estimated the sputtering rates and also simulated the evolution of the surface group composition over time. It was found that the bottom surface can be selectively cleared of the surface groups when using low acceleration voltages (60 kV). At high voltages (300 kV), the surface groups of the top surface also start to sputter. After clearing the surface groups, Ti atoms on the bare surface are relatively stable under the microscope. Since the bare surface is highly reactive, it can adsorb almost anything that is introduced afterwards, *e*.*g*., CO_2_,^37,38^ thus paving a way for engineering of the MXene surface. On the other hand, this also suggests that any residual gases in the chamber are likely to stick to the surface and hinder the imaging. Our simulated TEM images revealed that it might be possible to identify the degree of cleaning, *i*.*e*., whether there are surface groups or not, but the identification of the type of surface group is not possible based only on the intensity. However, the O groups lead to contraction of the lattice and shortening of the Ti–Ti bonds that is likely to be observable. In addition, we propose that graphene encapsulation could provide a viable pathway for protecting the MXene layers from electron beam damage during imaging, except for H which can be sputtered out through the graphene sheets. Finally, we evaluated the sputtering thresholds for five other MXenes using the formation energies of unrelaxed defects and found them to be close to those found for Ti_3_C_2_ and we thus believe that our findings for Ti_3_C_2_ are also largely valid for MXenes more generally.

## Conflicts of interest

The authors declare no conflict of interest.

## Supplementary Material

NA-003-D0NA00886A-s001
