# Supplementary material for: First principles study of the stability of MXenes under an electron beam
Source: Nanoscale Adv. 2021 Feb 17;3(7):1934–41. doi: 10.1039/d0na00886a (PMC9418968; doi:10.1039/d0na00886a)
Supplement: NA-003-D0NA00886A-s001 [file NA-003-D0NA00886A-s001.pdf]

Electronic Supplementary Material (ESI) for Nanoscale Advances.
This journal is © The Royal Society of Chemistry 2021

Supplementary information for "First principles study of stability of MXenes under electron beam"

Rina Ibragimova[1], Zhong-Peng Lv[1], and Hannu-Pekka Komsa[1,2]
[1] *Department of Applied Physics, Aalto University, Finland* and
[2] *Microelectronics Research Unit, University of Oulu, Finland*
(Dated: January 5, 2021)

I. SIMULATIONS OF KNOCK-ON MECHANISM

The system is first equilibrated for 1000 steps at about 200° C using a micro-canonical ensemble.

The maximum transferred momentum from a relativistic electron (upon backscattering and static lattice) is calculated from

$$E_{\max} = \frac{2E_e(E_e + 2m_0c^2)}{Mc^2} \quad \text{(S1)}$$

where E_e is the electron energy, m_0 is the electron mass, and M is the ion mass. [1] In this case, the momentum is parallel to the beam direction. At oblique angle θ to the beam direction, the transferred energy is $E_{\max}cos^2(\theta)$.

The collision of electron and ion is assumed to be instantaneous and thus we change the out-of-place component of the velocity of the impacted ion to $v = \sqrt{2E_{\max}/M}$. The kinetic energy of the ion is here at most tens of eVs and thus we can use the classical equation for the velocity.

We continue running MD until the impacted ion has either sputtered to the vacuum regions of the supercell or returned to the surface (to the original position or to another site). That is, we are determining threshold for sputtering, not for displacement. It is assumed that the diffusion of atoms on the surface is sufficiently fast that the displaced atoms

For calculating the McKinley-Feshbach cross section at finite T, we use the software provided in Ref. [2]. For the velocity distribution we use Maxwell-Boltzman distribution at room temperature.

II. RATE EQUATIONS

Let us denote the number of functional groups (per surface site) as N_O, N_{OH}, and N_F, and N_{vac} for empty site. Assuming these are the only possible ones, then $N_O + N_{OH} + N_F + N_{vac} = 1$. The rate equation for F is simply given by the corresponding sputtering rate r_F, but the numbers for O and OH are coupled since sputtering of H from OH give O group:

$$\frac{dN_F}{dt} = -r_F N_F \quad \text{(S2)}$$

$$\frac{dN_{OH}}{dt} = -r_{OH}N_{OH} - r_H N_{OH} \quad \text{(S3)}$$

$$\frac{dN_O}{dt} = -r_O N_O + r_H N_{OH} \quad \text{(S4)}$$

Using a shorthand notation $r' = r_H + r_{OH}$ the analytic solutions for these are:

$$N_F = N_F^0 e^{-r_F t} \quad \text{(S5)}$$

$$N_{OH} = N_{OH}^0 e^{-r' t} \quad \text{(S6)}$$

$$N_O = \frac{r_H N_{OH}^0}{r_O - r'} e^{-r' t} + (N_O^0 - \frac{r_H N_{OH}^0}{r_O - r'}) e^{-r_O t} \quad \text{(S7)}$$

III. SIMULATED ANNULAR BRIGHT FIELD IMAGES

Annular bright field (ABF) images for the same structures and energies as in Fig. 4 of the main paper are shown in Fig. S1. In addition, it shows how the images depend on the focus point.

[1] T. Susi, J. C. Meyer, and J. Kotakoski, Nature Reviews Physics **1**, 397 (2019).
[2] J. C. Meyer, F. Eder, S. Kurasch, V. Skakalova, J. Kotakoski, H. J. Park, S. Roth, A. Chuvilin, S. Eyhusen, G. Benner, A. V. Krasheninnikov, and U. Kaiser, Phys. Rev. Lett. **108**, 196102 (2012).

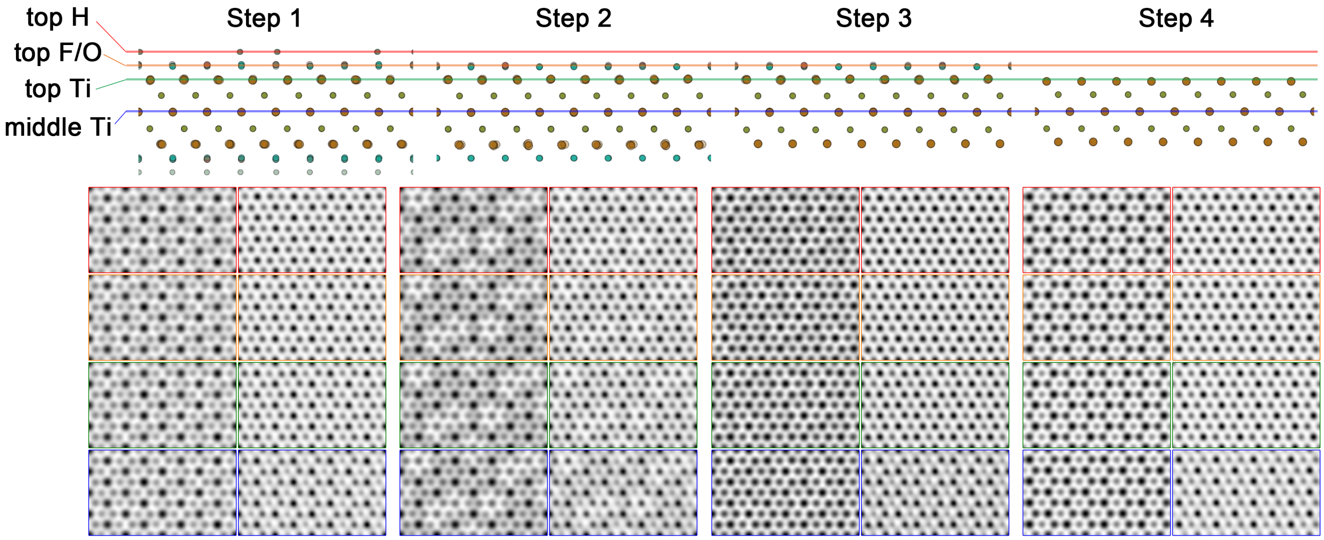

FIG. S1: Annular bright field images for the four structures considered in the main paper at 60 keV (left) and 300 keV (right) acceleration voltages and four different focus points (indicated by the box color).
